# Supplementary material for: Molecular Characterization of pBOq-IncQ and pBOq-95LK Plasmids of Escherichia coli BOq 01, a New Isolated Strain from Poultry Farming, Involved in Antibiotic Resistance
Source: Microorganisms. 2022 Jul 26;10(8):1509. doi: 10.3390/microorganisms10081509 (PMC9331969; doi:10.3390/microorganisms10081509)
Supplement: Supplementary file 1 [file microorganisms-10-01509-s001.zip › microorganisms-1815626-supplementary.pdf]

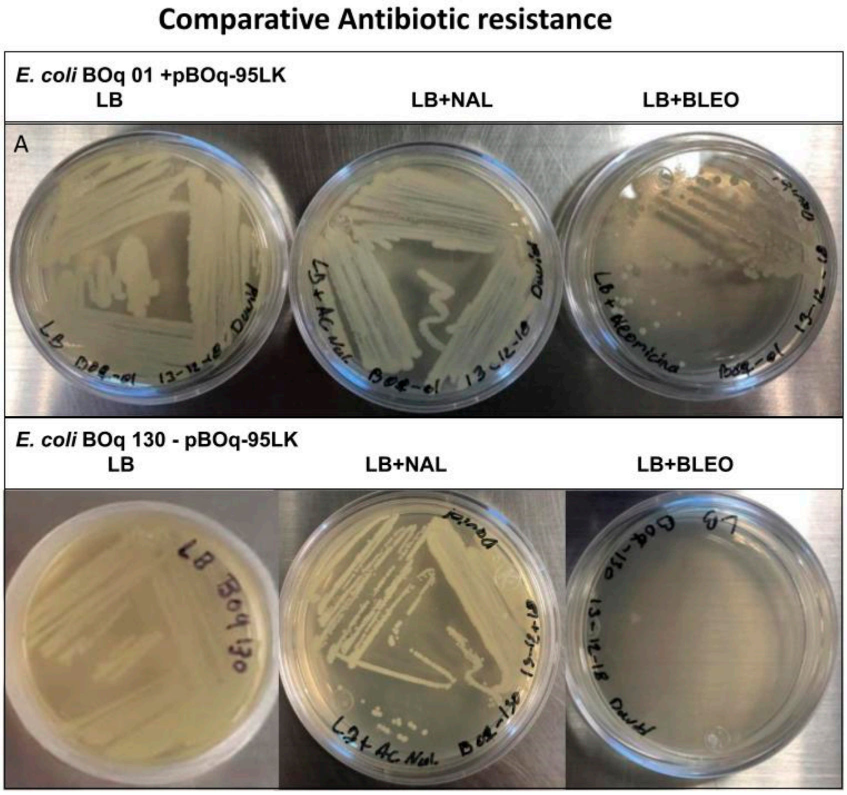

Supplemental Figure S1. The absence of pBOq-95LK increases susceptibility to bleomycin (A) *E. coli* strain BOq 01 with pBOq-95LK plasmid and (B) *E. coli* strain BOq 130 without pBOq-95LK plasmid growth in LB, LB with nalidixic acid (20 μg/ml) or bleomycin (20 μg/ml).

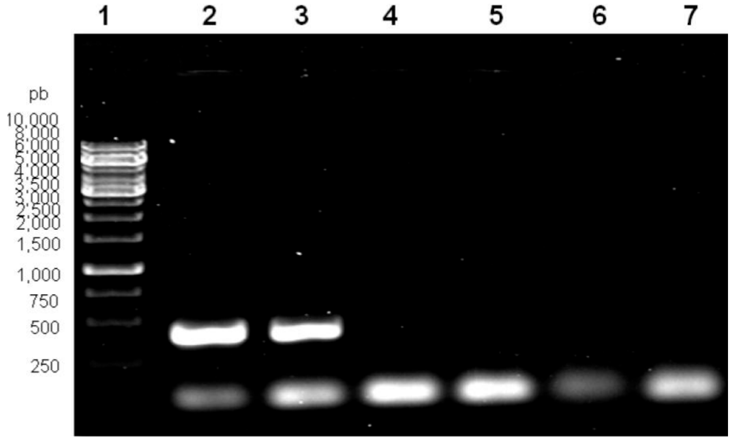

Supplemental Figure S2. Agarose gel electrophoresis (1%) of PCR in colony with the products of the fragment of the toxin-antitoxin system Phd-Doc present only in the pBOq-95LK plasmid. Line 1: DNA molecular size markers (1 kb), line 2-3: positive control *E. coli* BOq 01, lines 4-5: *E. coli* BOq 130 and lines 6 -7: negative control *E. coli* DH5α.

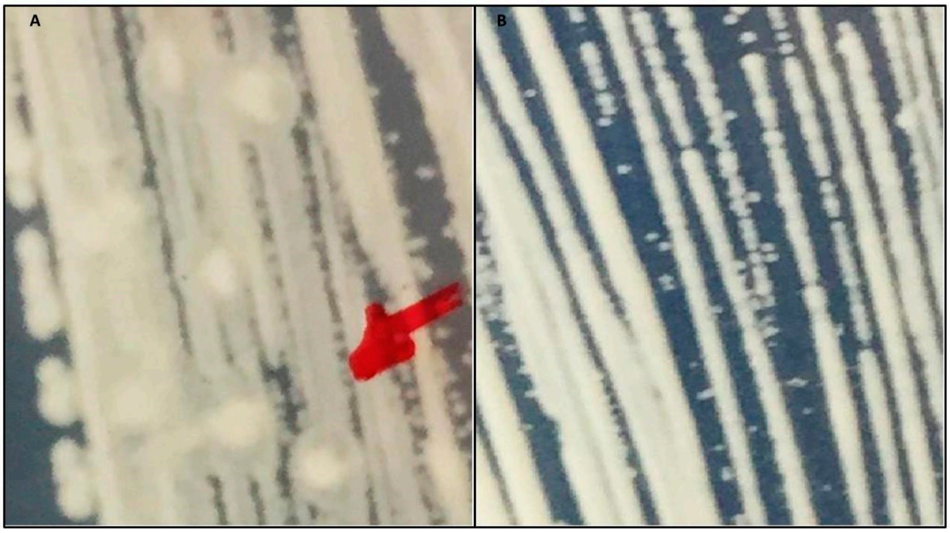

Supplemental Figure S3. Bacterial lysis plaque occurrence induced by UV light (A) *E. coli* strain BOq 01 with pBOq-95LK plasmid (B) *E. coli* strain BOq 01 without pBOq-95LK plasmid. The expanding translucent halo indicates bacterial lysis.

Supplemental Table S1. Plasmids obtained from the NCBI database used in this study for bioinformatic analysis, including their accession number for further reference.

Plasmid Name	Organism	Strain	Length (bp)	Query Cover	Identity to pBOq-95LK	Accession Number
pBOq-95LK	*E. coli*	BOq 01	95,980	100%	100%	QKQU02000012.1
pS51_2	*E. coli*	S51	98,216	85%	98%	CP015997.1
pO111_2	*E. coli* O111:H-	11128	97,897	82%	99%	AP010962.1
Plasmid_1	*Salmonella enterica* subsp. enterica serovar Typhi	ty3-243	97,394	83%	99%	LT905089.1
pMCR-1-P3	*E. coli*	IMP163	97,386	80%	98%	KX880944.1
p12579_1	*E. coli* O55:H7	RM12579	94,015	81%	99%	CP003110.1
p91	*E. coli*	127	91,199	86%	99%	CP023381.1
pFDAARGOS_3	*E. coli*	FDAARGOS_433	83,988	78%	98%	CP023896.1
p2014C-3084_1	*E. coli*	2014C-3084	78,854	68%	98%	CP027320.1
pMCR_SCKP-LL83	*K. pneumoniae*	SCKP-LL83	97,393	73%	99%	MF510496.1
pKLB08	*K. pneumoniae*	KLB08	96,564	68%	98.22%	MK112268.1
